# Protein–ligand pose and affinity prediction: Lessons from D3R Grand Challenge 3

**DOI:** 10.1007/s10822-018-0148-4

**Published:** 2018-08-20

**Authors:** Panagiotis I. Koukos, Li C. Xue, Alexandre M. J. J. Bonvin

**Affiliations:** Bijvoet Center for Biomolecular Research, Faculty of Science - Chemistry, Utrecht University, Padualaan 8, 3584 CH Utrecht, The Netherlands

**Keywords:** D3R, Drug Design Data Resource, Docking, Binding affinity, Ranking, HADDOCK

## Abstract

We report the performance of HADDOCK in the 2018 iteration of the Grand Challenge organised by the D3R consortium. Building on the findings of our participation in last year’s challenge, we significantly improved our pose prediction protocol which resulted in a mean RMSD for the top scoring pose of 3.04 and 2.67 Å for the cross-docking and self-docking experiments respectively, which corresponds to an overall success rate of 63% and 71% when considering the top1 and top5 models respectively. This performance ranks HADDOCK as the 6th and 3rd best performing group (excluding multiple submissions from a same group) out of a total of 44 and 47 submissions respectively. Our ligand-based binding affinity predictor is the 3rd best predictor overall, behind only the two leading structure-based implementations, and the best ligand-based one with a Kendall’s Tau correlation of 0.36 for the Cathepsin challenge. It also performed well in the classification part of the Kinase challenges, with Matthews Correlation Coefficients of 0.49 (ranked 1st), 0.39 (ranked 4th) and 0.21 (ranked 4th) for the JAK2, vEGFR2 and p38a targets respectively. Through our participation in last year’s competition we came to the conclusion that template selection is of critical importance for the successful outcome of the docking. This year we have made improvements in two additional areas of importance: ligand conformer selection and initial positioning, which have been key to our excellent pose prediction performance this year.

**Electronic supplementary material:**

The online version of this article (10.1007/s10822-018-0148-4) contains supplementary material, which is available to authorized users.

## Introduction

The Drug Design Data Resource (D3R) Grand Challenge (GC) of 2018 is the third iteration of the major docking competition organised by the D3R consortium [1, 2] and similarly to previous years, it has two goals. The first, is the assessment of the ability of docking algorithms to accurately predict the binding poses of a protein against a diverse set of small molecules, and the second, the evaluation of the performance of binding affinity prediction algorithms.

The protein which is the focus of the pose prediction assessment is Cathepsin S—a member of the Cathepsin family. Cathepsins are proteases that are classified in three groups depending on the makeup of their catalytic site, with Cathepsin S being a member of the most populated group—cysteine proteases [3]. Its involvement in MHC class II antigen presentation is well established. Given that role, it should come as no surprise that it has been implicated in many pathological conditions such as cancer and diabetes. More recently it has been investigated for its role in pain perception [4] and cardiovascular and kidney [5] disease. It has long held an interest for the pharmaceutical industry [6] as evidenced by the plethora (more than 50 at time of writing) of human Cathepsin S structures with a bound ligand, that have been deposited in the Protein Data Bank (PDB) [7] over a time period that spans 15 years.

In addition to the Cathepsin S-centric assessment, which also includes a binding affinity prediction component, binding affinity prediction approaches are evaluated in four subchallenges that focus on kinases. Kinases catalyse the process of phosphorylation through which a phosphate group is covalently bound to a protein substrate. Their role in cell signalling has been well understood for decades and they are involved in many aspects of cell differentiation and growth [8]. They are a primary target for cancer-related drug development [9].

Through our participation in last year’s GC [10] we came to the conclusion that template selection is of critical importance for the successful outcome of the docking. This year we have made improvements in two additional areas of importance: ligand conformer selection and initial positioning. The impact of this is reflected in our improved performance in GC3, the results of which are presented and discussed here.

## Materials and methods

HADDOCK (High Ambiguity Driven DOCKing) is our information-driven docking platform [11, 12]. For an introduction to HADDOCK and small molecule docking please review the contribution we made to last year’s special issue on the D3R GC [10]. The main conclusion from our participation in last year’s competition was that protein template selection is of crucial importance for the successful outcome of the docking. We used the protocol we came up with last year to select protein templates for this year’s competition as well. We made improvements to the ligand conformer selection and placement protocols. Similar to last year, all new and untested parts of the protocol were benchmarked on existing protein–ligand complexes extracted from the PDB.

In a departure from previous years, this year’s competition is further divided in five subchallenges. Subchallenge 1 is the equivalent of the GC of previous competitions and has a pose and binding affinity prediction component. Subchallenges 2–5 only have a binding affinity component. We participated in subchallenges 1 and 2.

### Subchallenge 1

This challenge focused on Cathepsin S. For the first part of the challenge—pose prediction—we had to predict the binding pose of Cathepsin S against a set of 24 small molecules that were known to bind to it. There is a cross-docking stage, during which the structures of the target proteins are not known and a self-docking stage for which the bound protein structures—but not those of the compounds—are known. The organisers provided us initially with SMILES strings for the small molecules and the FASTA sequence of the protein, and for the self-docking stage with the coordinates of the bound receptor for each ligand. Additionally, two publicly available structures of the protein with a dimethylsulfoxide (DMSO) molecule and a sulfate ion (SO_4_) placed in the binding pocket were circulated to the participants because the aforementioned molecules were detected in some of the crystal structures. For the binding affinity prediction component of the challenge we had to rank the binding affinities of 136 compounds against the protein.

#### Protein template selection

**Fig. 1:**
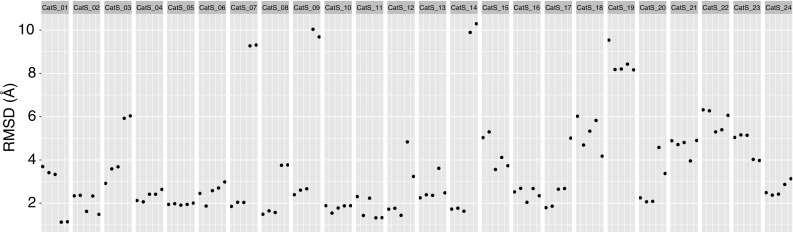
Heavy-atom RMSD values of the cross-docking models from the reference structures. Every point corresponds to one model with five models per target. The models are ranked by HADDOCK score with the highest scoring ones being on the left of every block

This part of the protocol, as well as the reasoning behind it, are described in greater detail in our previous work and so will only be covered briefly. Using the provided FASTA sequence, we identified structures of Cathepsin S that had been deposited in the PDB. We filtered the results and kept only those structures where the protein was complexed with a non-covalently bound ligand, thus identifying 36 templates. We then proceeded to compare the crystallographic ligands to the target compounds using as a similarity measure the Tanimoto distance, as implemented in the fmcsR and chemmineR packages [13, 14]. In this way, we selected one protein template for each of the 24 target compounds, by identifying the template with the highest similarity ligand. The similarities of the crystallographic ligands to the prediction set compounds are shown in S.I. Fig. 1.

For the self-docking challenge, we used the provided crystallographic structures retaining crystallographic waters and DMSO (target 14) or sulphate (targets 2, 17, 20, 22, 24 and 24) molecules.

#### Ligand preparation

Three-dimensional (3D) conformations of the ligands were generated with OpenEye OMEGA (v20170613) [15] using the SMILES strings as input. For every molecule, we sampled up to 500 conformers. We used the TanimotoCombo metric, as implemented in OpenEye ROCS [16], to compare the generated conformers to their respective crystallographic ligand in the identified templates (see “Protein template selection”). The TanimotoCombo metric combines shape and chemical similarity and allows us to select the conformers whose shape and chemical features resemble that of the crystallographic ligands. The top 10 scoring conformers were selected for ensemble docking. Each conformer was superimposed onto the crystallographic ligand in the template using the shape toolkit of the OpenEye suite.

This protocol was benchmarked with existing Cathepsin S-ligand structures identified in the PDB. This allowed us to evaluate the impact our choices had on the quality of our poses. We used four Cathepsin S structures (PDBids: 3IEJ, 3KWN, 3MPE, 3MPF) [17–19] and their respective ligands. After selecting the protein template based on the protocol described in “Protein template selection”, we selected the ligand conformers by their TanimotoCombo score and after superimposing them to the site of the crystallographic ligand, proceeded to refine them (see “Docking” below).

For the self-docking challenge, we superimposed the protein template identified during the cross-docking challenge on the prediction set crystallographic structure. That allows us to superpose the generated conformers on the crystallographic ligand which is situated in the active site of the prediction set crystallographic structure because of the first superposition.

#### Docking

We refined the ensemble of ligand conformations superimposed on their respective protein templates using the water refinement protocol of HADDOCK. All hydrogen atoms were kept (by default HADDOCK removes the non-polar hydrogens to save computing time). Since the ligand conformations were selected based on their similarity to the closest identified template (see above) and superimposed onto the ligand in the selected template, no exhaustive search was performed. Instead the initial poses were only subjected to a short energy minimization in which only interface residues were treated as flexible, followed by the explicit water refinement stage of HADDOCK. For this the system is solvated using an 8 Å shell of TIP3P [20] water molecules. The water refinement protocol consists of a first heating phase (100 MD integration steps at 100, 200, and 300 K) with weak position restraints on all atoms except those which belong to the side-chain of residues at the interface. The interface is defined as the set of residues whose atoms are within 5 Å of any atom of any binding partner. The second MD phase consists of 2500 integration steps at 300 K with positional restraints on all non-hydrogen atoms excluding the interface residues. The number of MD steps was doubled compared to HADDOCK’s default value (1250) because this yielded higher quality structures during our benchmarking with the four PDB structures described in “Ligand preparation”. The last cooling phase, consists of 500 integration steps at 300, 200 and 100 K, respectively, during which positional restraints are only used for the backbone atoms of the non-interface residues. A 2 fs time-step is used throughout the protocol for the integration of equation of motions. The number of water refined models was set to 200. We also modified the default HADDOCK scoring function for the refinement stage by halving the weight of the electrostatic energy term:

$$HADDOC{K_{score}}=1.0 \times {E_{vdw}}+0.1 \times {E_{elec}}+1.0 \times {E_{desolv}}+0.1 \times {E_{AIR}}$$

This adjustment was motivated by internal benchmarking our group has performed on small molecule–protein complexes (data not shown). This scoring function is used to rank the generated models. The various terms are the intermolecular van der Waals (E_vdw_) and electrostatic (E_elec_) energies calculated with the OPLS force field and an 8.5 Å non-bonded cutoff [21], an empirical desolvation potential (E_desolv_) [22] and the ambiguous interaction restraints energy (E_AIR_) [11]. Note that in this case, since only refinement was performed without any restraints to drive the docking, E_AIR_ is effectively 0.

For the self-docking challenge, we follow the same protocol as for the cross-docking one, keeping all crystallographic waters and fixing the conformation of the protein, with the additional change of instructing HADDOCK to write PDB files containing the solvent molecules (water) present during the refinement stage.

#### Binding affinity

The binding affinity predictions are evaluated in two stages. The first stage takes place before the structures of the complexes (protein and ligand) are released by the organisers, which means that either only ligand information is used, or models of the complexes, and the second after, which allows participants to make use of the newly available structural information.

For the first stage, we submitted both ligand-based and structure-based rankings and for the second only a structure-based one. Both approaches are described in detail in our previous D3R paper [10]. In short, the structure-based approach consists of the PRODIGY [23] method adapted for small molecules and trained on the 2P2I dataset [24] which makes use of the following function to score protein–ligand complexes by binding affinity:

$$\Delta{{\text{G}}_{{\text{score}}}}=0.343794 \times {{\text{E}}_{{\text{elec}}}} - 0.037597 \times {\text{A}}{{\text{C}}_{{\text{CC}}}}+0.138738 \times {\text{A}}{{\text{C}}_{{\text{NN}}}}+0.160043 \times {\text{A}}{{\text{C}}_{{\text{OO}}}} - 3.088861 \times {\text{A}}{{\text{C}}_{{\text{XX}}}}+187.011384$$

where ${E_{elec}}$ is the intermolecular electrostatic energy calculated by the water refinement protocol of HADDOCK (see “Docking”) and $A{C_{CC}}$, $A{C_{NN}}$, $A{C_{OO}}$ and $A{C_{XX}}$ are the counts of atomic contacts between carbon–carbon, nitrogen–nitrogen, oxygen–oxygen and all other atoms and polar hydrogens between the protein and the ligand, within a distance cut-off of 10.5 Å. We used the mean ${\triangle G_{{\text{score}}}}$ of the top 10 models of the water refinement (see “Docking”) to rank the compounds.

The ligand-based approach rests on the hypothesis that similar ligands complexed to our proteins of interest should have similar binding affinities. Using the BindingDB database [25] we identified 1839 compounds bound to Cathepsin S with IC50 values. We calculated the similarity of the prediction set to the training set using the Atom Pair measurement as a similarity measure. The similarity matrices of the BindingDB set were used to train a Support Vector Regression model with the libSVM library for MatLab [26] that was, in turn, used to predict the binding affinities of the prediction set.

#### Analysis

Fitting and RMSD calculations for generating the figures were performed using the McLachlan algorithm [27] as implemented in the program ProFit (http://www.bioinf.org.uk/software/profit/) from the SBGrid distribution [28].

### Subchallenge 2

Subchallenge 2 only had a binding affinity component. The participants had to predict binding affinities for three protein targets—the kinases vEGFR2, JAK2-SC2 and p38-α—and sets of 85, 89 and 72 compounds respectively. Some of the compounds were shared between the three targets. The organisers provided SMILES strings for all compounds along with FASTA sequences of the proteins.

For this challenge, we only submitted ligand-based binding affinity rankings. The method is the same as the one described in “Binding affinity” section for subchallenge 1. The only difference was the training data availability. Using BindingDB we identified 7049, 4582 and 4563 compounds with IC50 binding affinity measurements for the vEGFR2, JAK2-SC2 and p38a kinases respectively.

After the binding affinity rankings were released by the organisers, it quickly became apparent that for all three targets, the compounds could be classified into binding and non-binding sets since most compounds had the maximum detectable binding affinity of 10 µM. This prompted the organisers to alter the way the challenge would be evaluated into a classification and regression problem, where the identification of the binding set (compounds with a Kd < 10 µM) would be treated as a classification problem and the ranking of the binding compounds by binding affinity as a regression problem.

## Results and discussion

### Subchallenge 1

#### Pose prediction

The binding pose prediction was evaluated for the cross- and self-docking experiments. Our performance in the cross-docking experiment in terms of RMSD of the five submitted poses is shown in Fig. 1.

**Fig. 2:**
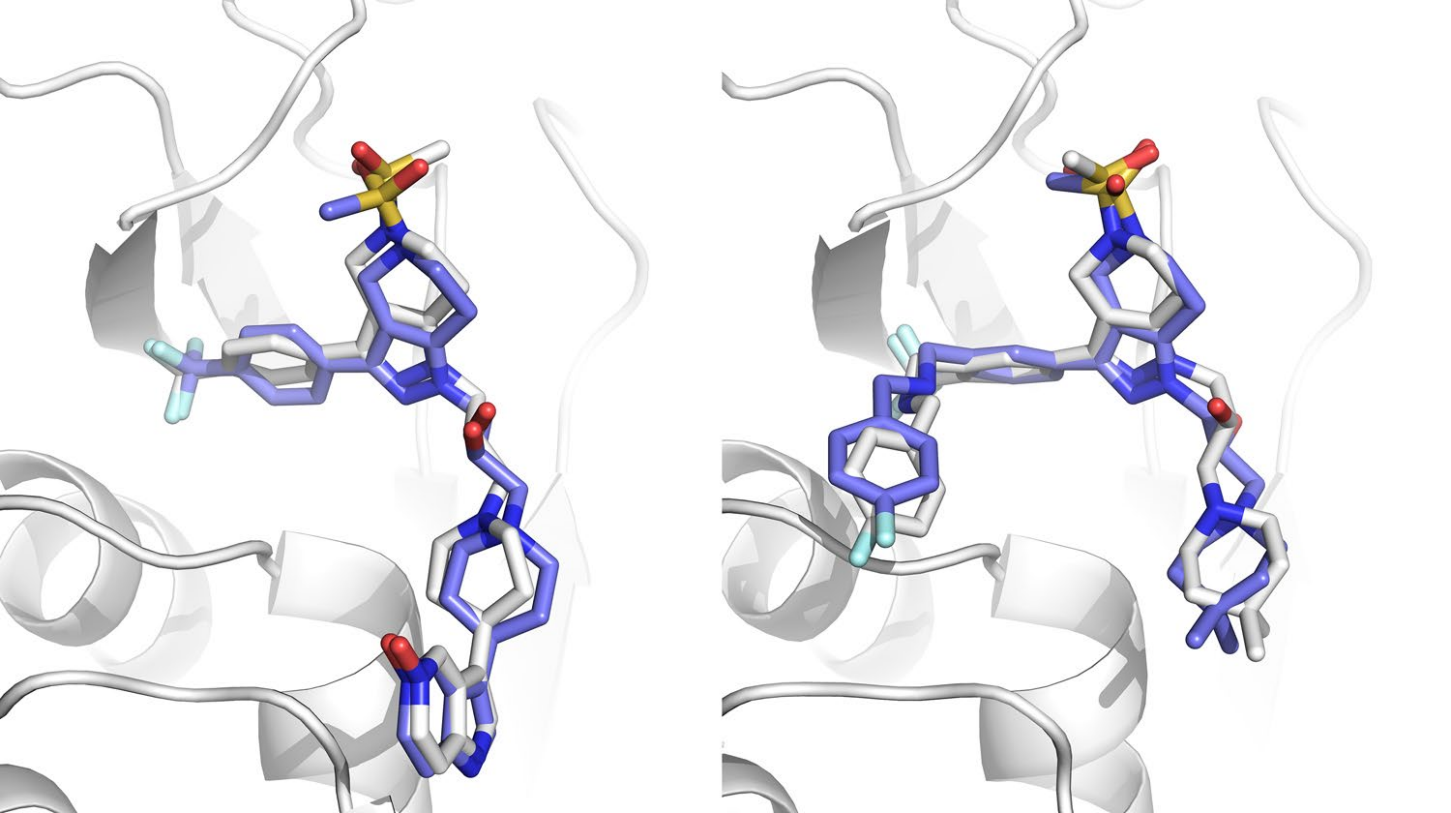
Superpositions of HADDOCK models on reference structures. Left: model 5 from target 1 (1.1 Å). Right: model 1 from target 8 (1.5 Å). The reference protein structure is shown in cartoon representation in white. The compounds are shown in stick representation in white and blue for the reference and model molecules respectively. Figure created with PyMOL [29]

This analysis was carried out by superposing the interface areas of the models and their respective reference structures and calculating the heavy-atom RMSD (excluding any halogen atoms) of the compounds. The mean RMSD values across all models and targets for this experiment are 3.04 ± 2.03 Å, whereas for the self-docking experiment, the values improved to 2.67 ± 1.63 Å. Figure 2 highlights some of our top predictions.

**Fig. 3:**
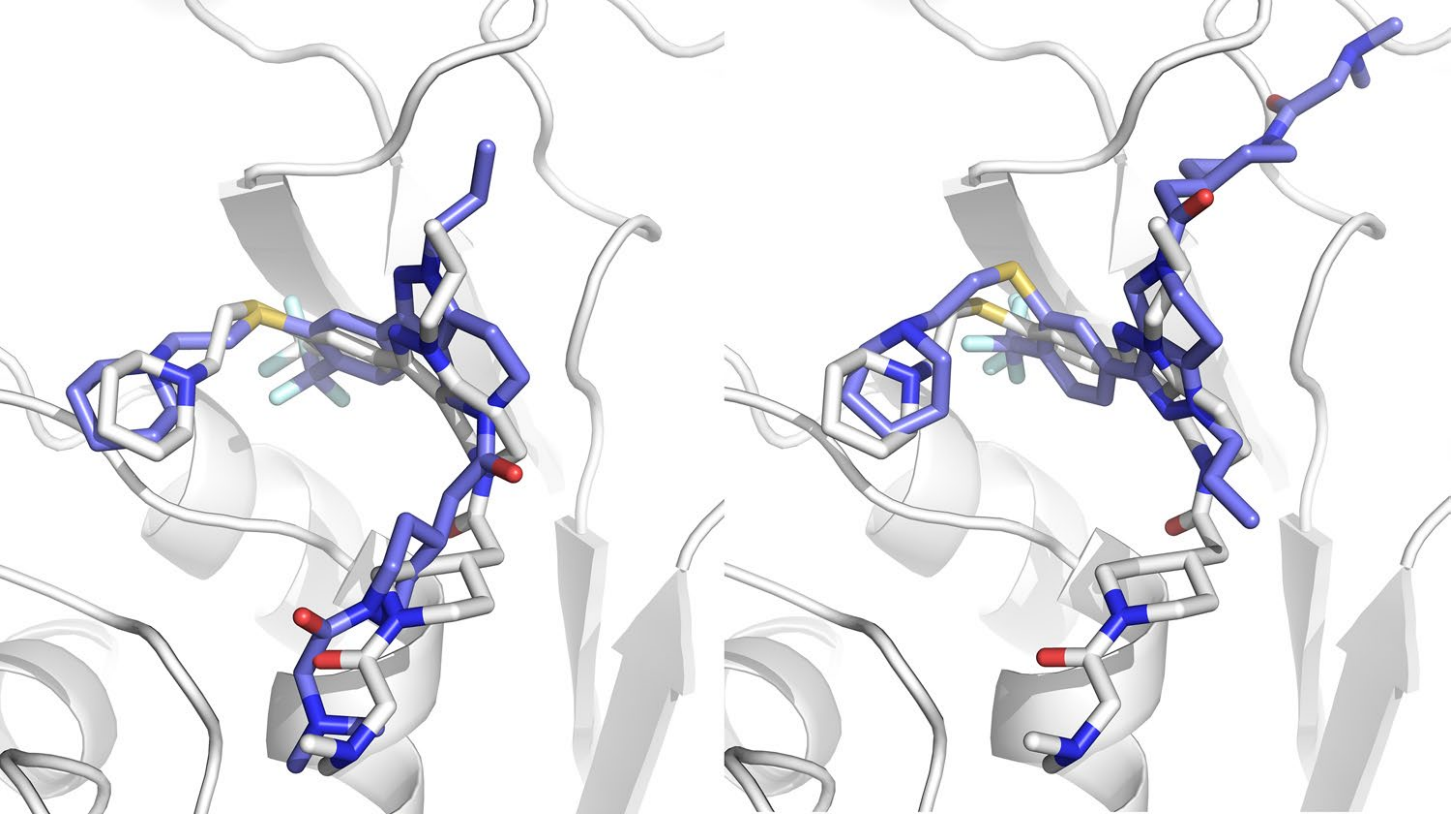
Superpositions of HADDOCK models on reference structures. Left: model 1 from target 7 (1.85 Å). Right: model 5 from same target (9.31 Å). Our scoring function can distinguish the near native model from the wrong one. The difference between the two molecules is a single torsional angle that has been rotated ~ 180°. Figure created with PyMOL

At least one of the 5 models submitted was of acceptable quality (RMSD ≤ 2.5 Å) in 17 of the 24 targets (71% success rate top5). Our scoring function is thus able to correctly rank the near-native solutions near the top as can be seen in S.I. Fig. 2. If one considers only the top-ranked pose, the performance remains impressive with 15 out of 24 targets with an acceptable quality model (63% success rate top1). Figure 3 shows the difference between the top and bottom ranked models for target 7. Despite these excellent results, there is still room for improvement, especially in scoring: if we only consider the targets for which we generated at least one acceptable model (17 out of 24), the top-scoring pose corresponds to the best pose in 5 of the 17 targets (29%). For the remaining 12 targets, the average difference between the top scoring and best poses is 0.55 ± 0.71 Å and 0.45 ± 0.61 Å for the cross- and self-docking experiments respectively.

**Fig. 4:**
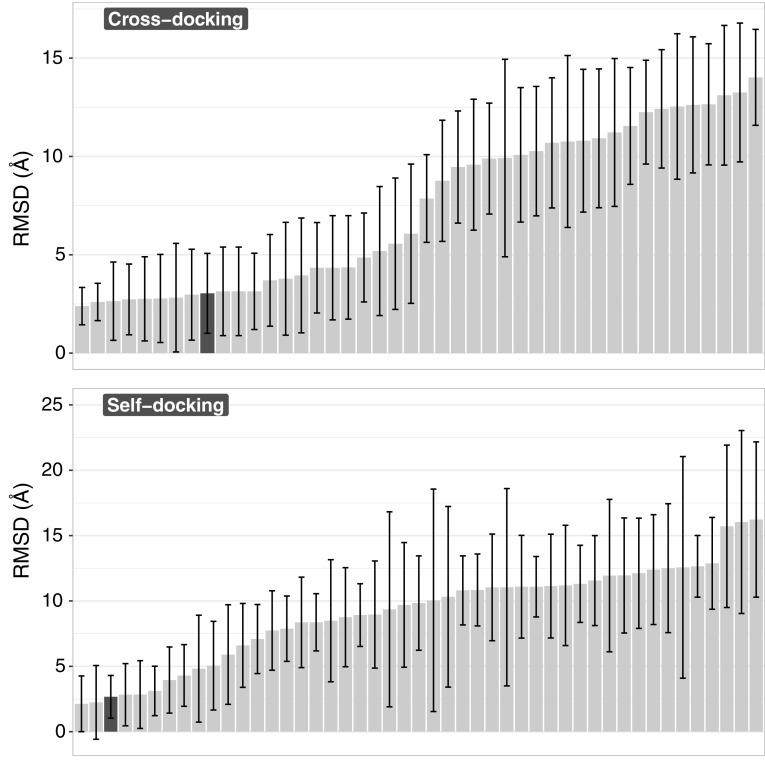
Heavy-atom RMSD values averaged over all models and all targets. Top: cross-docking experiment. Bottom: self-docking experiment. Every bar corresponds to a single submission. The error bars indicate the standard deviation of the mean RMSD. HADDOCK submission is represented by the dark-grey bar in both panels

**Fig. 5:**
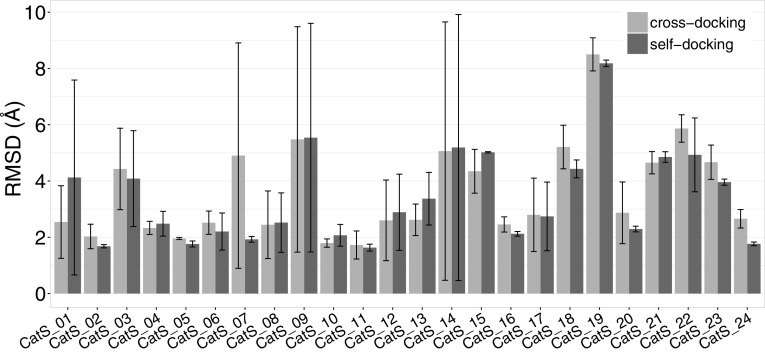
Comparison between the performance of HADDOCK in the cross-docking and self-docking stages. Every set of bars corresponds to the average heavy-atom RMSD of all five models for a target, with the light- and dark-grey coloured bars corresponding to the cross- and self-docking experiments respectively. The error bars indicate the standard deviation of the mean RMSD

The performance of HADDOCK relative to the other participants for both experiments can be seen in Fig. 4. Note that if we would only consider one submission (the best) per group our rank would be 6th for the cross-docking experiment (top panel in Fig. 4). Our performance in the two experiments (cross- vs. self-docking) is broken down by target in Fig. 5, revealing that our protocol is not very sensitive to the starting template. In most cases only rather small improvements in terms of RMSD are obtained when starting from the bound receptor including water. The single target for which we observe a significant deviation in the self-docking results compared to the cross-docking ones is the first one (see Fig. 5). The average RMSD for that target is 2.54 ± 1.29 Å and 4.13 ± 3.46 Å for cross- and self-docking experiments respectively. Model 5 of the self-docking experiment submission is mostly responsible for this significant change, since its RMSD is > 10. This is a repetition of what is shown in Fig. 3, with one of the models (model 5 in both cases) which has a torsional angle that is rotated by 180° compared to the rest of the submitted models and the reference structure.

#### Binding affinity prediction

**Fig. 6:**
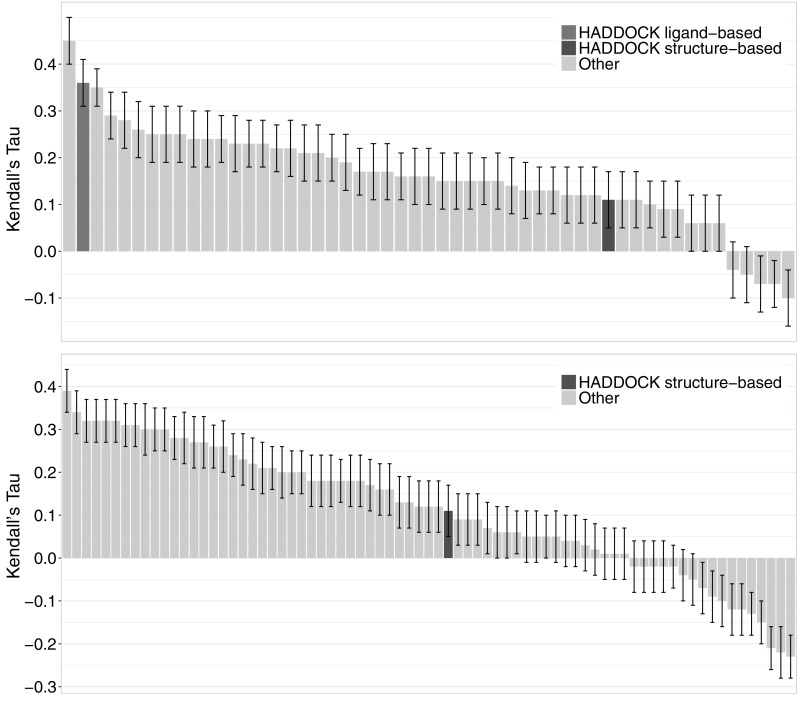
Ranking of the binding affinity predictions for Cathepsin S by correlation. Top: stage (1). Bottom: stage (2). Every bar corresponds to one submission with our ligand-based submission having a medium and the structure-based one a dark grey colour in both panels

Binding affinity predictions were performed in two stages—one before the organisers released the poses to the participants and one after. We participated in stage 1 with both ligand-based and structure-based approaches, while for stage 2 we only submitted a structure-based ranking. Figure 6 shows our performance compared to all participants.

These results were rather surprising: The structure-based approach which was one of the top performers in last year’s competition failed to produce an accurate ranking of the compounds, while our ligand-based predictor now performs as one of the best (even if the quality of the prediction is still limited). There was also no improvement for the structure-based ranking between stages 1 and 2 in contrast to GC2 where we noticed a significant improvement when using the crystallographic poses for ranking the compounds. One explanation for this could be that, compared to last year, we already had better quality poses for most of the targets for stage 1. On the other hand, our simple machine learning-based ligand-based approach is not only the most accurate ligand-based approach with a Kendall’s Tau of 0.36 but the third most accurate method for both stages, behind only the top performing structure-based approaches.

### Subchallenge 2

**Fig. 7:**
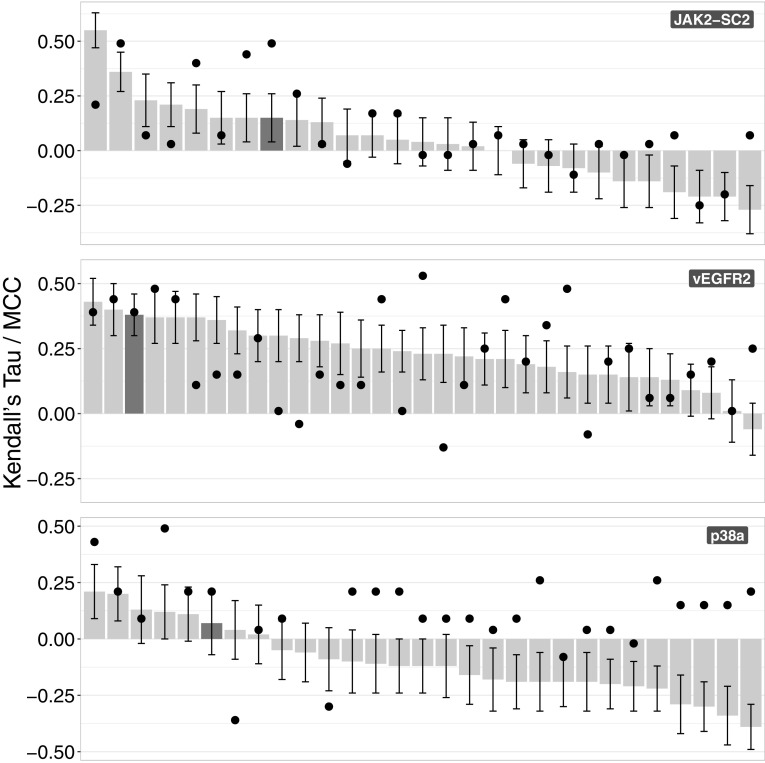
Binding affinity prediction correlation coefficients. Top: JAK2-SC2. Middle: vEGFR2. Bottom: p38a. The bars and the corresponding error bars represent the Kendall’s Tau correlation between the binding affinity predictions and the binding set for every target. The black circles correspond to the Matthews Correlation Coefficient which was used to assess the accuracy of the classification of the compounds into binding and non-binding. The dark grey bars and their corresponding circles represent our submissions

This challenge revolved around kinase binding affinity prediction. As was mentioned in “Materials and methods” section, this is a regression-classification problem. The overall results can be seen in Fig. 7.

Despite the fact that our approach wasn’t trained with classification in mind, the classification performance is better than that of the regression. Specifically, the Matthews Correlation Coefficient values are 0.49, 0.39 and 0.21 respectively for JAK2-SC2, vEGFR2 and p38a (see S.I. Fig. 3 for the classification rankings). The respective Kendall’s Tau correlations are 0.15, 0.38 and 0.07. As is evident from the plot the two correlation metrics are not correlated. This means that an algorithm that accurately identifies the binders and non-binders does not necessarily rank the binders accurately. The performance differences cannot be accounted for by the difference in training set size, since we identified roughly the same number of compounds for JAK2-SC2 and p38a. Additionally, vEGFR2 had the biggest training set size but that is not translated into better performance for the classification or the regression.

## Conclusions

GC3 has allowed to implement the lessons that we learned by participating in GC2 and further experiment with additional optimisations. The conclusions that we can draw with regards to the pose prediction challenges are the following:

1. Selecting the protein templates accurately has the largest effect on the outcome of the docking. By identifying templates that already have a ligand bound to them and selecting the one that is most similar to the prediction compounds, we are ensuring a protein binding interface that is highly compatible with the prediction compound. This removes the need for extensive sampling of the protein interface or ensemble docking. Moreover, this approach seems to be robust to low similarity (see S.I. Fig. 1) compounds. The majority of template ligands identified have a Tanimoto similarity of < 0.6.
2. Selecting the ligand conformations. Identifying structures with existing compounds has the additional benefit that they can be used to select the compound structures to be used during docking. Generating 3D models of compounds from 2D information entails generating hundreds of conformers. By comparing the shape and chemical similarity of the conformers to existing compound structures we can reduce the number of conformers needed during docking and ensure the starting conformations are closer to the experimental structures.
3. Making use of the template information by positioning the conformers in the binding interface. This last observation is only relevant for molecular simulation codes that, like HADDOCK, randomise the relative orientation and position of the partners prior to docking. We can use shape similarity to position the ensemble of conformers at the binding site and bypass the first two stages of HADDOCK (rigid-body energy minimisation and flexible refinement by simulated annealing in torsion angle space) and directly refine the complexes using a longer version of our water-refinement protocol.

The applicability of our approach was demonstrated by its performance, with mean RMSD values of 3.04 Å and 2.67 Å for the cross-docking and self-docking experiments respectively. Our overall success rate when considering the top1 and top5 poses is 63% and 71%, respectively. These results place us as the 6th and 3rd best performers for the two challenges respectively.

The binding affinity experiments present a greater challenge to the community as whole. Despite our competitive rankings in the classification as well as the regression challenges, it appears that reliable binding affinity predictors are still not within grasp. This holds true for both ligand and structure-based approaches. However, the surprisingly good classification results (especially given that the algorithm was optimised for regression rather than classification problems) make us optimistic that this can be improved in the future.

## Additional Information

The data and code used to train the ligand-based binding affinity predictor and rank the compounds are freely available on GitHub, together with our in-house scripts developed during our participation in the last two GC competitions. These can be accessed at following URL: https://github.com/haddocking/D3R-tools.

## Electronic supplementary material

Below is the link to the electronic supplementary material.

Supplementary material 1 (PDF 281 KB)
