# Supplementary material for: Protein–ligand pose and affinity prediction: Lessons from D3R Grand Challenge 3
Source: J Comput Aided Mol Des. 2018 Aug 20;33(1):83–91. doi: 10.1007/s10822-018-0148-4 (PMC6373529; doi:10.1007/s10822-018-0148-4)
Supplement: Supplementary file 1 — Supplementary material 1 (PDF 281 KB) [file 10822_2018_148_MOESM1_ESM.pdf]

Supplementary material for

Protein-ligand pose and affinity prediction. Lessons from D3R Grand Challenge 3.

Panagiotis I. Koukos[1], Li C. Xue[1], Alexandre M.J.J. Bonvin[1*]

[1]Bijvoet Center for Biomolecular Research, Faculty of Science - Chemistry, Utrecht University, Padualaan 8, Utrecht, 3584CH, the Netherlands

*To whom correspondence should be addressed: a.m.j.j.bonvin@uu.nl, phone: +31 30 2533859

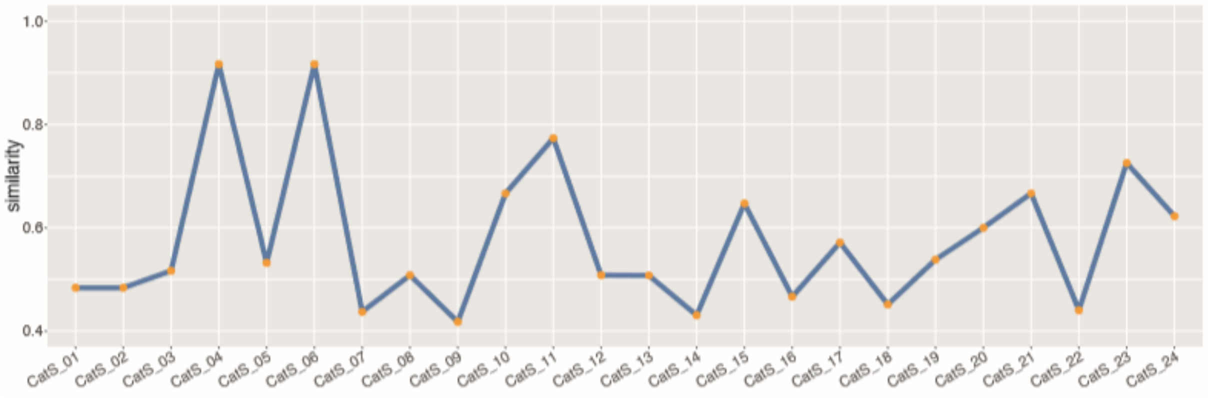

Figure S1: Tanimoto Similarity of the prediction set compounds to the most similar crystallographic ligand of the ones identified in the PDB. A value of 1 indicates perfect similarity and a value of 0 perfect dissimilarity.

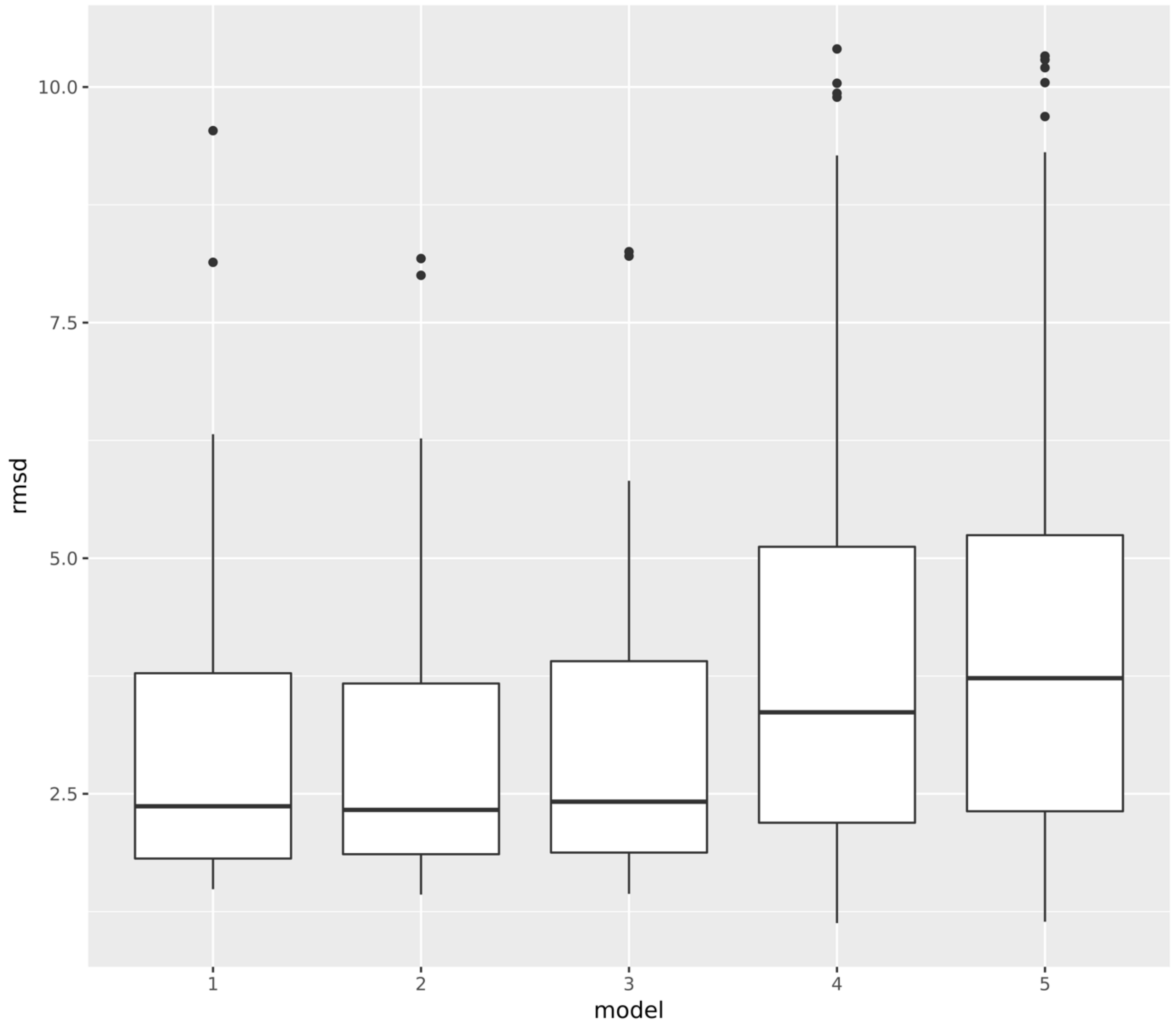

Figure S2: Heavy-atom RMSD values grouped by ranking. The models in group 1 were the ones that were ranked at the top by HADDOCK and the ones in group 5 the ones ranked at the bottom.

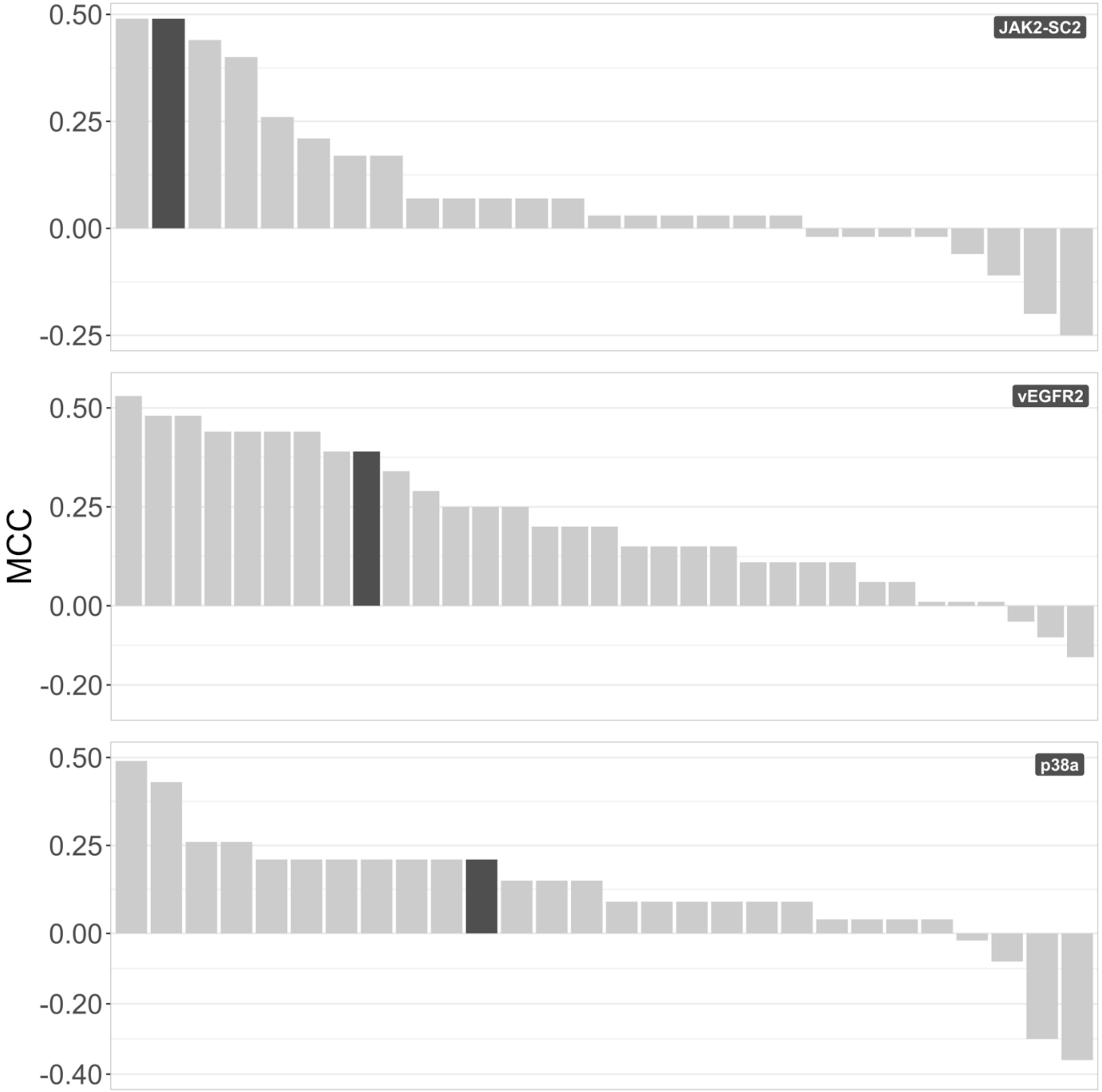

Figure S3: Binding affinity prediction classification performance. Top: JAK2-SC2. Middle: vEGFR2. Bottom: p38a. The bars indicate the Matthews Correlation Coefficient for every submission. The dark grey bars correspond to our submissions.
